# A multi-class defect detection method for substations based on the improved YOLOv10n

**DOI:** 10.3389/frai.2026.1759740

**Published:** 2026-05-13

**Authors:** Long Huang, Kangning Li, Tianren Fu, Jinlei Zhu, Yunhao Zhong, Xingfei Wang

**Affiliations:** Guangzhou Power Supply Bureau, Guangdong Power Grid Co., Ltd., CSG, Guangzhou, Guangdong, China

**Keywords:** content-guided attention, detail information extraction convolution, substation defect detection, weighted hybrid fusion pyramid network, YOLOv10n

## Abstract

**Introduction:**

Ensuring the stable operation of power substations is critical for maintaining the reliability of the electrical grid. However, automated inspection of substation equipment remains challenging because multi-class defects are often small, visually blurred, and located in complex backgrounds

**Methods:**

To improve the localization accuracy of small defects with fuzzy features, this paper proposes YOLO-SMALLNET, an improved defect detection algorithm based on YOLOv10n. First, a Detail Information Extraction Convolution module is used to replace the strided convolution modules in the baseline network to preserve fine-grained information during downsampling. Second, a low-level feature fusion detection layer is introduced to reduce small-target feature loss. Third, a Weighted Hybrid Fusion Pyramid Network is adopted to optimize multi-scale feature integration. Finally, a Content-Guided Attention mechanism is integrated to enhance critical defect information while suppressing background noise.

**Results:**

Experimental results show that, compared with the baseline model, YOLO-SMALLNET improves Precision, Recall, mAP@0.5, and mAP@0.5:0.95 by 7.3, 8.2, 3.9, and 3.3%, respectively.

**Discussion:**

The proposed method effectively reduces false detections and missed detections of small defect regions and is suitable for real-time automated inspection of substation equipment.

## 1 Introduction

The electric power industry constitutes the backbone of modern infrastructure, with substations acting as pivotal hubs for voltage transformation and power distribution (Chen et al., 2021). The operational reliability of substation equipment, ranging from transformers and insulators to switchgear, directly dictates the stability and safety of the entire power grid (Zhu et al., 2023). Traditionally, the maintenance and inspection of these facilities relied heavily on manual labor, a process that is not only time-consuming and labor-intensive but also fraught with safety risks for personnel (Wang J. et al., 2024). With the rapid development of smart grid technologies, the industry has increasingly shifted toward automated inspection systems utilizing inspection robots and Unmanned Aerial Vehicles (UAVs). These systems generate massive amounts of visual data, necessitating robust computer vision algorithms to automatically identify and categorize equipment defects in real-time (Liu et al., 2022).

Despite significant advancements in object detection technologies, the reliable identification of multi-class defects in substation environments remains a formidable challenge (Wang et al., 2025; Zhao et al., 2024). Unlike standard datasets with prominent objects, substation imagery is characterized by complex backgrounds, varying lighting conditions, and extreme scale variations. Defects such as fine cracks on insulators, corrosion spots on metal fittings, or oil leakage traces often occupy a negligible portion of the image and exhibit blurred features (Qian et al., 2024). These “small targets” are easily overwhelmed by background noise or lost during the feature extraction process of deep neural networks (Park et al., 2020; Wang Z. et al., 2024). Similar challenges of detecting anomalous patterns in visually complex scenes have been observed in other domains, such as abnormal activity recognition in video surveillance (Kumar et al., 2024) and moving object detection under dynamic conditions (Sengar and Mukhopadhyay, 2017a; Sengar and Mukhopadhyay, 2017b). Consequently, standard detection models frequently suffer from high missed detection rates and poor localization precision when applied to these subtle yet critical defects.

In recent years, deep learning-based object detection algorithms have become the mainstream approach for defect detection (Zhang et al., 2024a; Ou et al., 2022). Among them, the YOLO (You Only Look Once) series has gained widespread popularity due to its superior balance between detection speed and accuracy. Specifically, the recently introduced YOLOv10 has pushed the boundaries of real-time detection by eliminating the need for Non-Maximum Suppression (NMS) and optimizing model architecture for efficiency (Chu et al., 2025). However, when applied to the specific domain of substation defect detection, even advanced baselines like YOLOv10n face limitations. The standard strided convolution operations used for downsampling often discard high-frequency information essential for describing the boundaries of small, blurry defects. Furthermore, the feature pyramid structures in standard models may not adequately fuse low-level texture information with high-level semantic information, leading to insufficient feature representation for minute targets (Zhang et al., 2021; He et al., 2025; Liao et al., 2025).

To address these limitations and enhance the precision of defect localization in substations, this paper proposes an improved algorithm named YOLO-SMALLNET. The proposed method builds upon the YOLOv10n architecture but introduces specific structural modifications tailored for feature-blurred small target detection. To prevent the loss of fine-grained details, we design and implement a Detail Information Extraction Convolution (DIEConv) module. By replacing the standard strided convolution modules in the baseline network with DIEConv, we ensure that detailed spatial information is preserved during the feature extraction phase. This is particularly crucial for identifying defects where the visual cues are subtle and easily degraded by resolution reduction.

Furthermore, to bolster the network’s sensitivity to small targets, we introduce a low-level feature fusion detection layer. In deep convolutional networks, small objects often disappear in the deeper layers due to repeated downsampling. By explicitly integrating lower-level features that retain higher spatial resolution, our model significantly reduces the feature loss associated with small targets. In the neck of the network, we deploy a Weighted Hybrid Fusion Pyramid Network (WHFPN). Unlike standard fusion techniques that treat all input features equally, WHFPN assigns learnable weights to different feature maps, allowing the network to prioritize the most relevant information scales for multi-class defect detection.

Finally, the complexity of substation backgrounds, filled with gantries, wires, and vegetation, requires the model to focus strictly on the defect areas. To achieve this, we incorporate a Content-Guided Attention (CGA) mechanism. The CGA module dynamically weighs the importance of spatial and channel-wise features, effectively guiding the network’s focus toward the defect characteristics while suppressing irrelevant background interference. This enhances the information fusion efficiency between feature layers and improves the extraction of key diagnostic information.

The remainder of this paper is organized as follows: Section II reviews related work in object detection and substation defect detection. Section III presents the detailed methodology of the improved YOLOv10 model. Section IV describes the experimental setup and analyzes the results. Finally, Section V concludes the paper and outlines future research directions.

## 2 Related work

The application of deep learning techniques to substation equipment defect detection has become a critical research area, driven by the increasing demand for automated monitoring in power infrastructure. Intelligent vision-based approaches are progressively replacing traditional manual inspections, offering continuous, objective, and comprehensive monitoring capabilities (Xu et al., 2023; Saberironaghi et al., 2023; Li et al., 2025; Zou et al., 2025). The primary challenges in this domain stem from the diverse nature of electrical equipment, the variety of potential defect types, and the demanding operational environments of substation facilities (Jiao et al., 2025).

The YOLO family of algorithms has gained significant traction in industrial defect detection due to its excellent balance of accuracy and computational efficiency. Beyond the YOLO family, attention-based architectures have also demonstrated strong potential in detecting small targets against complex backgrounds. For instance, RepSE-CBAMNet (Khan et al., 2025) employs hybrid attention mechanisms to enhance focus on diagnostically critical regions in medical imaging, while RepVGG-GELAN (Balakrishnan and Sengar, 2024) improves feature extraction efficiency through structural backbone reparameterization. These works provide supporting evidence that attention mechanisms and backbone redesign are effective strategies for enhancing detection sensitivity in challenging scenarios. Researchers have adapted YOLO architectures for substation monitoring through various innovations.

Early adaptations focused on backbone optimization and hybrid methodologies. Pian et al. (Qian et al., 2022) developed LFF-YOLO with ShuffleNetv2 as the backbone, achieving notable performance but showing diminished effectiveness for defects with significant scale variations. Dong et al. (2023) explored a hybrid approach combining big data analytics with traditional detection frameworks, leveraging text mining to identify recurring defect patterns.

Subsequent research emphasized small defect detection and feature enhancement. Wu et al. (2023) addressed poor small-target recognition in complex substation scenarios by improving YOLOv5 with deformable convolution, BiFPN-based adaptive weighted fusion, and an additional small-object detection layer. Zhang et al. (2024b) further advanced the field with a YOLOv9-based model integrating multi-scale attention and inner-SIoU loss to improve small-target detection and convergence.

More recent developments have shifted toward lightweight, deployable models using YOLOv10 and YOLOv11 architectures. Meng et al. (2025) proposed comprehensive modifications to YOLOv10n to address adverse weather conditions, achieving 96.64% detection accuracy and demonstrating the potential of modern lightweight architectures for field deployment.

Despite these advances, several critical limitations persist: (1) Most approaches excel at detecting specific defect types but struggle with comprehensive multi-class scenarios. (2) The fundamental challenge of accurately detecting miniaturized defects against complex backgrounds remains inadequately addressed. (3) Existing feature extraction mechanisms often fail to capture the fine-grained details necessary to distinguish between subtle defect variations. (4) Limited generalization capability across different substation configurations and equipment types restricts the broader applicability of current solutions. Our work aims to address these specific gaps by creating a robust, multi-scale detection framework optimized for small defects.

## 3 Methodology

### 3.1 Overall architecture of YOLO-SMALLNET

**Figure 1:**
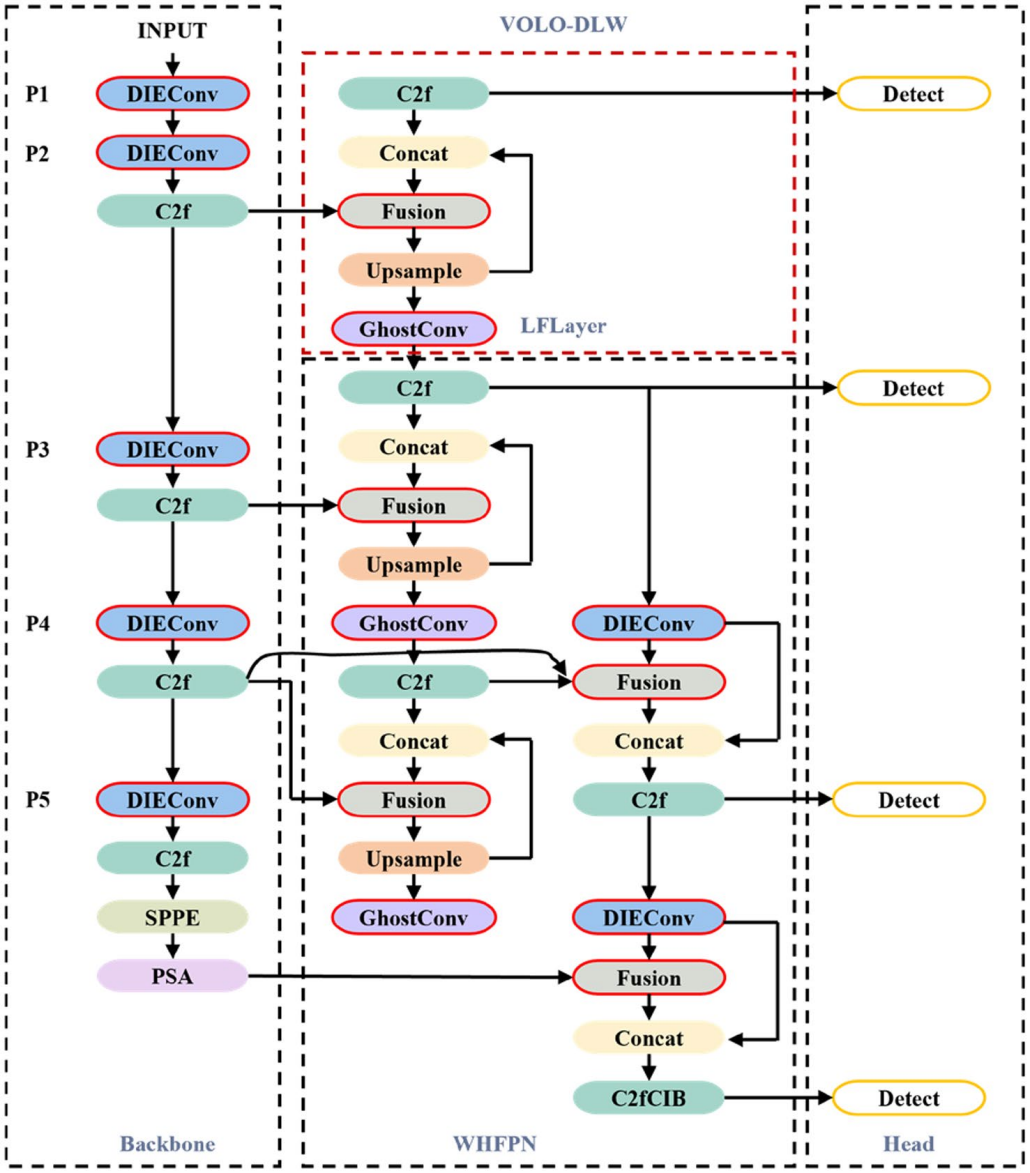
The overall architecture of the proposed YOLO-SMALLNET network.

Based on the characteristics of the defect regions that need to be detected in substation equipment, this paper proposes YOLO-SMALLNET, an algorithm model specifically designed for the localization and detection of feature-blurred small targets. We select YOLOv10n, which has the fewest parameters among the high-performing YOLOv10 series, as our baseline model, and redesign both the backbone and neck networks (Wang et al., 2026). The overall architecture of YOLO-SMALLNET is illustrated in Figure 1.

The modules highlighted in red boxes in the figure represent the new modules added or replaced in the baseline model. Specifically, the DIEConv module replaces the strided convolution modules in the baseline model structure. The GhostConv module is an additionally inserted non-strided convolution module that serves to adjust the number of channels. The LFLayer represents the newly added low-level feature fusion detection layer. The Fusion module is the CGA (Content-Guided Attention) feature fusion module proposed in this paper as part of the Weighted Hybrid Fusion Pyramid Network structure.

The architectural design philosophy behind YOLO-SMALLNET stems from the unique challenges posed by substation defect detection. Traditional object detection networks often sacrifice spatial resolution for semantic richness through aggressive downsampling operations. While this approach works well for detecting large, visually distinct objects, it proves inadequate when dealing with minute defects that occupy only a few pixels in the image space. Our proposed architecture addresses this limitation through three key innovations: preserving fine-grained spatial information during feature extraction, incorporating multi-scale feature fusion with explicit low-level feature integration, and implementing content-aware attention mechanisms to guide the network’s focus toward defect-relevant information.

The network takes an input image and processes it through the modified backbone network equipped with DIEConv modules. These modules ensure that even after multiple stages of feature extraction, the detailed spatial information necessary for identifying small, blurry defects is retained. The extracted features are then fed into the WHFPN structure in the neck, where features from different scales are intelligently fused using learnable weights guided by content-specific attention mechanisms. Finally, the detection heads operating at multiple scales, including the newly introduced low-level detection layer, produce the final detection results with improved localization precision for small targets.

### 3.2 Design of detail information extraction backbone network

Convolutional Neural Networks (CNNs) are widely applied in computer vision tasks, and in the YOLOv10 algorithm, CNNs form a core part of its architecture. In traditional CNN designs, strided convolution is typically used for downsampling operations to extract spatial features, with common kernel sizes being 3×3 or larger. Strided convolution is implemented by setting a stride greater than 1 in the convolution operation, meaning that the convolutional kernel moves multiple pixels at a time rather than sliding pixel by pixel across the input feature map (Li et al., 2024).

For instance, when the stride is set to 2, the kernel moves 2 pixels each time, and only one out of every two pixels in the input feature map is covered by the convolutional kernel, thereby halving the spatial dimensions of the output feature map. Because strided convolution skips a portion of the input data, this leads to some important local information potentially not being captured. Although this feature downsampling, can aggregate contextual information and achieve the goal of reducing spatial dimensions, it comes at the cost of losing detailed information, making it challenging for the model to recognize and learn features of small targets.

The pooling layers in traditional designs similarly reduce the resolution of feature maps and decrease computational load. However, during this process, information about small objects may be excessively compressed or completely lost, leading to degraded detection performance. Therefore, when using these modules for downsampling, the detailed information of defect regions in substation equipment images is inevitably lost, affecting the network’s ability to extract fine-grained features from small-scale defects. Moreover, the defect regions occupy an extremely small area in the captured images, and the uneven illumination caused by varying outdoor lighting conditions and equipment occlusion further necessitates that the network extract more detailed information to improve recognition capability for small targets (Zhang et al., 2025).

**Figure 2:**
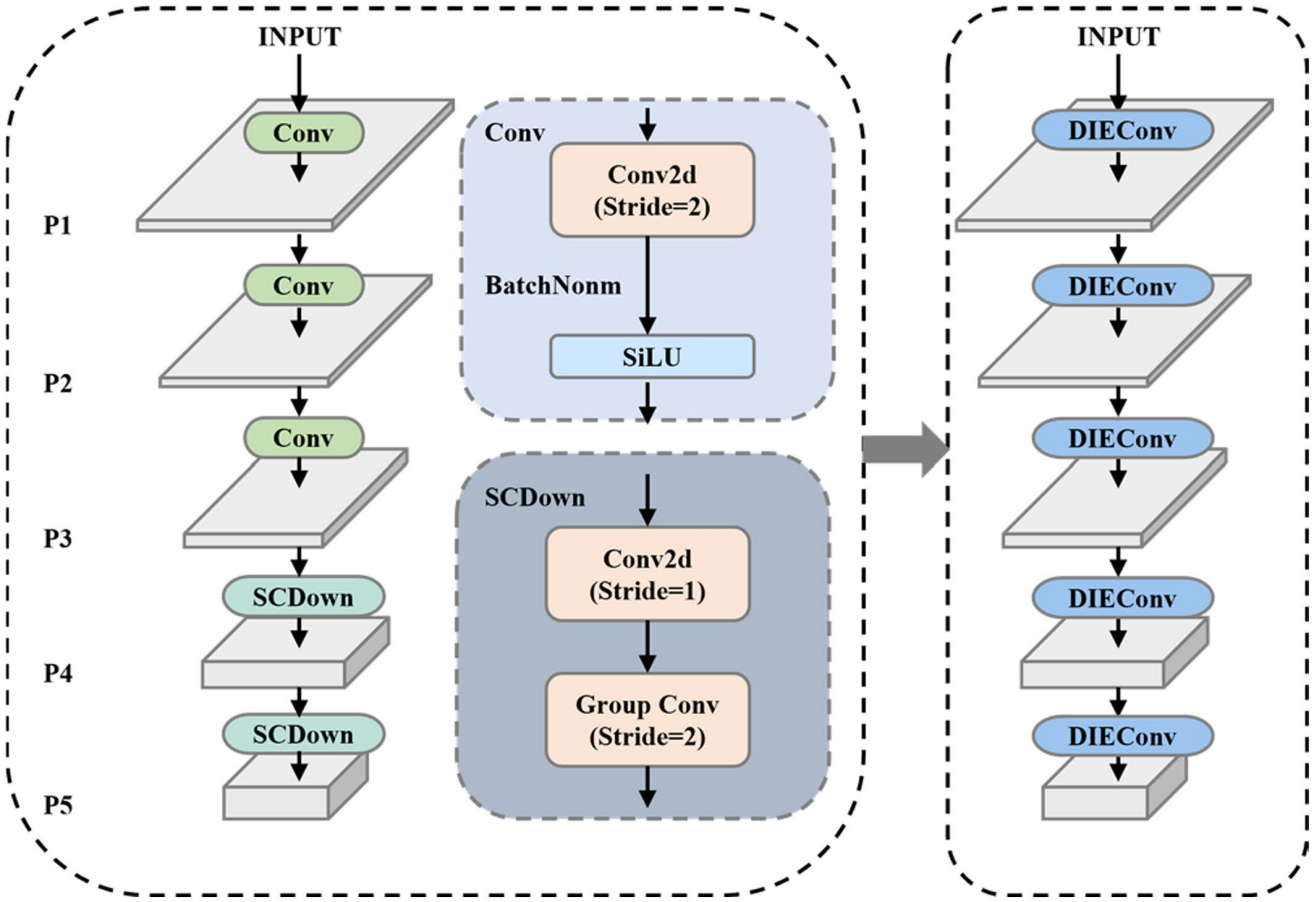
Backbone network structure diagram.

To address these issues, this paper employs the Detail Information Extraction Convolution (DIEConv) module in the backbone network of YOLOv10 to replace traditional strided convolution modules, improving small target detection performance by preserving fine-grained information and avoiding excessive compression of image features. The replacement process is shown in Figure 2.

**Figure 3:**
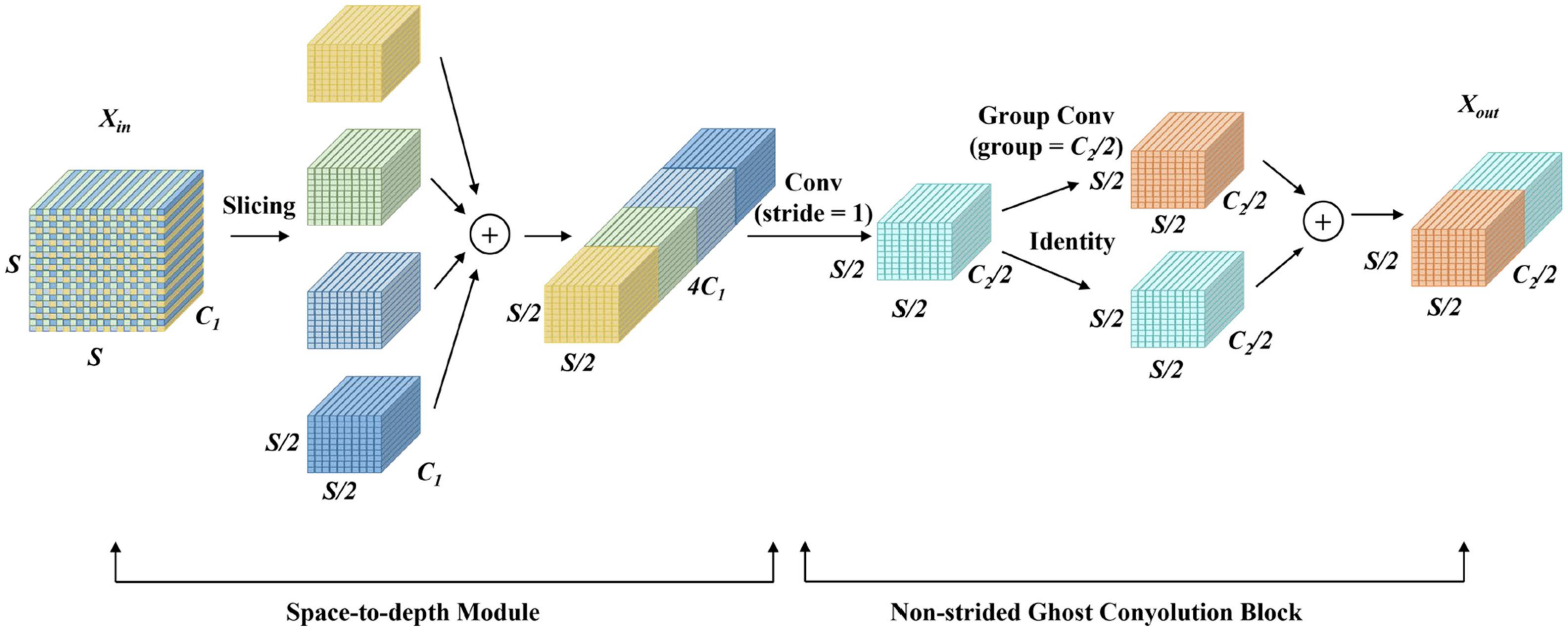
DIEConv module structure diagram.

The DIEConv module primarily consists of a Space-to-Depth Module and a Non-strided Ghost Convolution Block, replacing all strided convolution blocks in the YOLOv10 backbone network. The Space-to-Depth module first performs pixel-wise partitioning and rearranges the pixels of each block into depth channels, achieving spatial compression of the input feature map. This reorganization not only halves the spatial dimensions of the feature map but also preserves all original information of the processed pixels, effectively avoiding the detail loss that traditional strided convolution may cause during spatial compression. The main structure of the module is illustrated in Figure 3.

Let $X_{\text{in}}$ denote the input feature map to the DIEConv module, with *S* representing the spatial dimension (height and width) of the input feature map, and $C_{1}$ denoting the number of input channels. Let $X_{\mathrm{spd}}$ represent the output feature map of the Space-to-Depth module, with $C_{2}$ being the number of output channels, and $X_{\text{out}}$ denoting the output feature map of the DIEConv module.

The Space-to-Depth module first performs a feature map slicing operation, formulated in Equation 1:


$$f_{h,w}=X[h:S:\text{scale},w:S:\text{scale}]$$ (1)

where X represents the input feature map, *h* and *w* are the starting indices for height and width, respectively. *S* is the size of the input feature map, and scale is the stride of the slicing operation. When scale = 2, the four sliced feature maps are defined in Equation 2:


$$\left\{ \begin{array}{l} f_{0,0}=X[0:S:2,0:S:2]\\ f_{0,1}=X[0:S:2,1:S:2]\\ f_{1,0}=X[1:S:2,0:S:2]\\ f_{1,1}=X[1:S:2,1:S:2] \end{array}\right.$$ (2)

These sliced feature maps and their channel-wise concatenation is shown in Equation 3:


$$X_{\mathrm{spd}}=\text{Concat}[f_{0,0}:f_{0,1}:f_{1,0}:f_{1,1}]$$ (3)

where Concat[⋅] represents the channel-wise concatenation operation. The Space-to-Depth module preserves detailed information while reducing the spatial dimensions of the feature map.

The subsequent channel-reduction operation in the Non-strided Ghost Convolution Block is given in Equation 4:


$$X_{\text{out}}=\text{Concat}[{\mathrm{GC}}_{5\times 5}(F_{C_{2}/2}^{1\times 1}(X_{\mathrm{spd}})):F_{C_{2}/2}^{1\times 1}(X_{\mathrm{spd}})]$$ (4)

where ${\mathrm{GC}}_{5\times 5}$ denotes a group convolution operation with a 5×5 kernel size, and $F_{C_{2}/2}^{1\times 1}$ represents a 1×1 convolutional transformation function with $C_{2}/2$ output channels. Through these operations, the DIEConv module achieves downsampling while maximally preserving all detailed information from the original image, without significantly increasing computational cost.

The effectiveness of DIEConv lies in its information-preserving downsampling strategy. Unlike traditional strided convolution that directly discards spatial information through subsampling, the Space-to-Depth operation systematically reorganizes spatial information into the channel dimension. This ensures that every pixel value from the input is retained in the output, albeit in a different organizational structure. The subsequent Ghost Convolution efficiently reduces the channel dimensionality while maintaining feature expressiveness through a split-transform-merge strategy, where only a portion of channels undergo expensive spatial convolution operations, and the remaining channels are generated through cheap linear operations.

### 3.3 Low-level feature fusion detection layer

In traditional YOLO series network designs, the Path Aggregation Feature Pyramid Network (PAFPN) adopts a structure of downsampling followed by upsampling and then downsampling again, combining skip connections to enhance information exchange between feature maps (Bai et al., 2025). Feature maps are divided into five levels, P1 through P5, based on their spatial reduction ratio relative to the input image (1/2, 1/4, 1/8, 1/16, and 1/32, respectively).

After multiple downsampling operations, some low-level features may gradually be lost. Although skip connections between downsampling and upsampling paths at the same hierarchical level help to some extent in recovering detailed information lost due to consecutive convolution and pooling operations, for extremely small targets, the original structure performs only two upsampling operations after five downsampling stages. This detail recovery remains insufficient, affecting the network’s detection performance.

**Figure 4:**
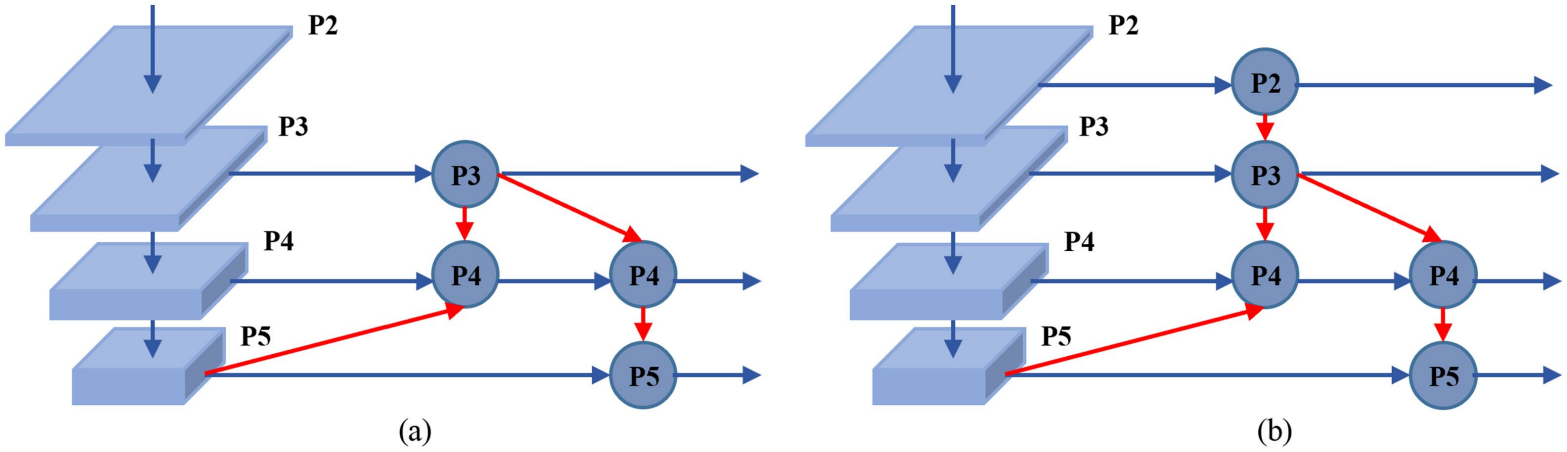
LFLayer principle schematic. **(a)** The original network structure of YOLOv10. **(b)** The network structure after adding LFLayer.

To address this issue, we introduce a low-level feature fusion detection layer in the neck network to preserve detailed features of extremely small targets. We fuse the P2 feature layer from the backbone network (with a downsampling factor of 2) and the P2 feature layer obtained by upsampling the P5 feature layer three times. This fused feature map is directly fed to the small target detection head. The purpose of this design is to enhance the model’s ability to locate and recognize objects of extremely small sizes by retaining sufficient low-level features. The principle of the low-level feature fusion detection layer is illustrated in Figure 4.

Let $P_{2}^{\text{backbone}}$ denote the P2 feature map from the backbone network, with spatial dimensions $H_{2}\times W_{2}$ and $C_{2}$ channels. Let $P_{5}^{\text{backbone}}$ represent the P5 feature map with dimensions $H_{5}\times W_{5}$ and $C_{5}$ channels, where typically $H_{5}=H_{2}/8$ and $W_{5}=W_{2}/8$ . The upsampling operation is formulated in Equation 5:


$$P_{5}^{\mathrm{up}3}={\text{Upsample}}_{8\times }(P_{5}^{\text{backbone}})$$ (5)

where ${\text{Upsample}}_{8\times }(\cdot )$ represents an 8× upsampling operation, typically implemented through bilinear or nearest-neighbor interpolation, bringing the spatial dimensions of $P_{5}^{\mathrm{up}3}$ to match those of $P_{2}^{\text{backbone}}$ .

The channel adjustment before fusion is formulated in Equation 6:


$$P_{5}^{\mathrm{adj}}=F_{C_{\text{target}}}^{1\times 1}(P_{5}^{\mathrm{up}3})$$ (6)

where $C_{\text{target}}$ is the target channel number for fusion, and $F_{C_{\text{target}}}^{1\times 1}(\cdot )$ denotes a 1×1 convolution operation that adjusts the channel dimension to $C_{\text{target}}$ . The resulting low-level feature fusion operation is defined in Equation 7:


$$P_{\mathrm{LF}}={\text{Conv}}_{3\times 3}(P_{2}^{\mathrm{adj}}+P_{5}^{\mathrm{adj}})$$ (7)

where ${\text{Conv}}_{3\times 3}(\cdot )$ denotes a 3×3 convolution for feature integration, and $P_{\mathrm{LF}}$ represents the low-level fusion feature map that serves as input to the small target detection head.

This strategy of combining low-level detailed features with high-level semantic features not only helps improve detection performance for small targets but also maintains computational efficiency to a certain extent. Through this method, the model can more accurately capture and recognize minute objects in images. The rationale is that low-level features, being closer to the input image, retain finer spatial information such as edges, corners, and texture patterns that are critical for localizing small defects. Meanwhile, the deeply upsampled high-level features provide semantic context that aids in distinguishing actual defects from background clutter. The element-wise addition operation allows the network to simultaneously leverage both information sources, creating a feature representation that is both spatially precise and semantically meaningful.

### 3.4 Weighted hybrid fusion pyramid network

Traditional YOLO series models employ the Path Aggregation Feature Pyramid Network (PAFPN) as their neck network. However, there is no information interaction between each layer of feature maps from the PAFPN and the backbone, which may result in the loss of some detailed features. The preservation of detailed features is particularly important for small target recognition.

Tan et al. proposed the Bi-directional Feature Pyramid Network (BiFPN), which adds cross-scale fusion layers compared to PAFPN, enabling top-down and bottom-up feature flow. By adding weights to each feature input, the feature fusion process is optimized, helping to retain more useful information. However, since BiFPN introduces dynamic weights that are optimized through backpropagation during network training, these learnable dynamic weights may increase the computational burden of the network. Additionally, the uncertainty in the magnitude of the original weights may lead to training instability in the early stages.

**Figure 5:**
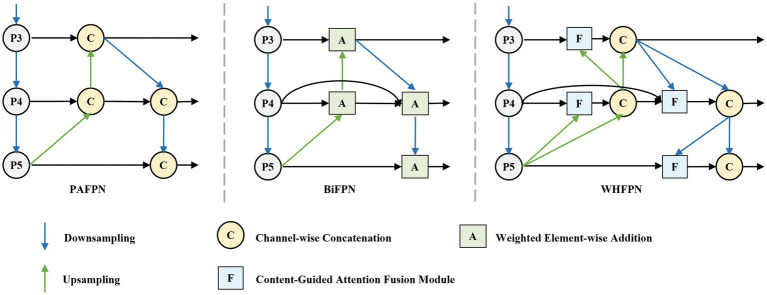
Comparison of PAFPN, BiFPN, and WHFPN structures.

To address these issues, we redesign the original neck network by introducing a feature propagation path from the backbone directly to the downsampling path, reducing the loss of small target features during propagation. We propose a feature fusion strategy combining the CGA mechanism. This strategy dynamically adjusts fusion weights by evaluating the content relevance of feature maps at each layer. Unlike traditional BiFPN, which uses fixed algorithms to assign weights, the CGA mechanism can adaptively allocate more weight to features that are more critical to the final detection task based on the specific content in the feature maps. This not only enhances the model’s detection capability for small targets but also strengthens the model’s expressiveness across various scales. The network structure is shown in Figure 5.

**Figure 6:**
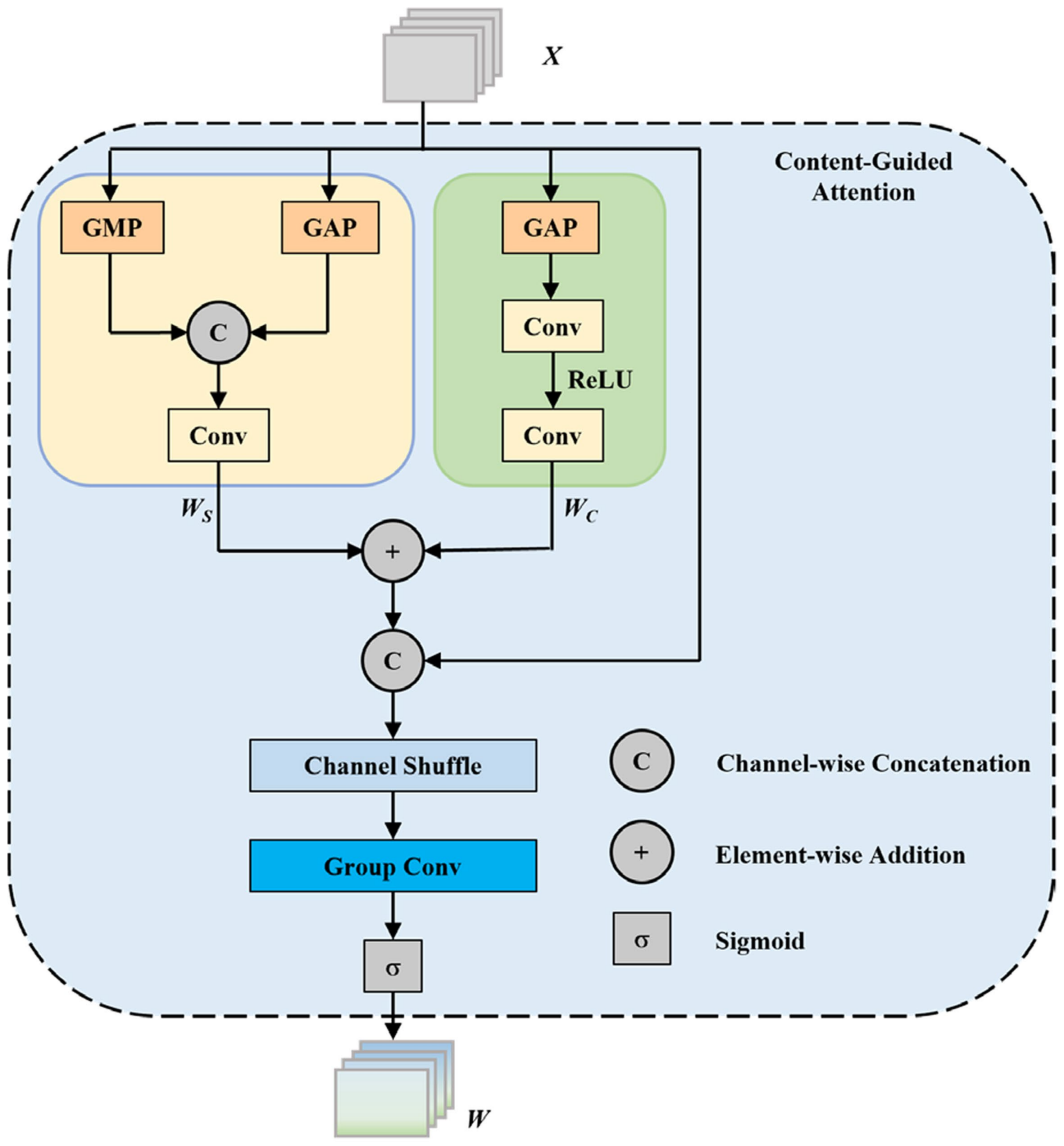
Content-guided attention (CGA) structure diagram.

The principle of the CGA mechanism is illustrated in Figure 6. Let X be the input feature map with dimensions H×W×C , where H and W denote spatial height and width, and C represents the number of channels. The channel attention and spatial attention feature extraction steps are formulated in Equations 8, 9, respectively.


$$W_{c}=F_{C}^{1\times 1}(\max(0,F_{C/r}^{1\times 1}(X_{\mathrm{GAP}}^{C})))$$ (8)

$$W_{s}=F_{C}^{7\times 7}([X_{\mathrm{GAP}}^{s},X_{\mathrm{GMP}}^{s}])$$ (9)

where $W_{c}\in {\mathbb{R}}^{1\times 1\times C}$ represents channel attention features, $W_{s}\in {\mathbb{R}}^{H\times W\times 1}$ represents spatial attention features, $F_{C}^{1\times 1}(\cdot )$ denotes a 1×1 convolution with C output channels, r is the channel reduction ratio (typically set to 16), max(0,⋅) represents the ReLU activation function, $X_{\mathrm{GAP}}^{C}\in {\mathbb{R}}^{1\times 1\times C}$ denotes global average pooling across spatial dimensions, and $X_{\mathrm{GAP}}^{s}\in {\mathbb{R}}^{H\times W\times 1}$ and $X_{\mathrm{GMP}}^{s}\in {\mathbb{R}}^{H\times W\times 1}$ represent global average pooling and global max pooling across channel dimensions, respectively. The concatenation operator [⋅,⋅] combines these two pooled features along the channel dimension.

Subsequently, the features from both dimensions are added and concatenated with the input X along the channel dimension. After a channel shuffle operation, the result passes through group convolution and a Sigmoid operation to obtain the fusion weight W is computed by Equation 10:


$$W=\sigma ({\mathrm{GC}}_{7\times 7}(\mathrm{CS}(\text{Concat}[X,W_{c}+W_{s}])))$$ (10)

where σ(⋅) represents the Sigmoid activation function that normalizes the output to the range [0, 1], ${\mathrm{GC}}_{7\times 7}(\cdot )$ denotes group convolution with a 7×7 kernel size (with the number of groups typically set to C/8, CS(⋅) represents the channel shuffle operation that promotes information flow across different channel groups, and Concat[⋅] denotes channel-wise concatenation.

**Figure 7:**
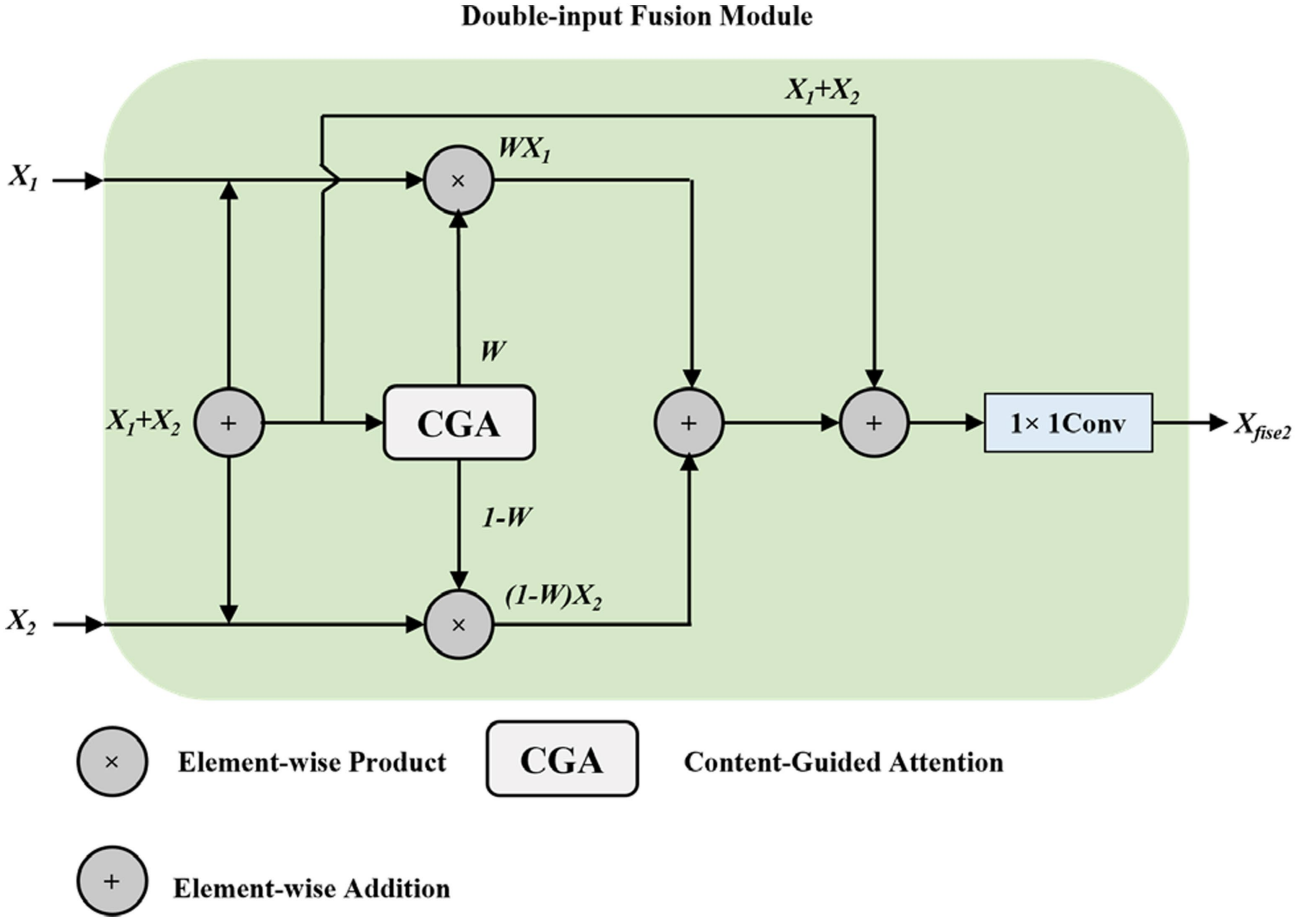
Two-input fusion module structure diagram.

The Content-Guided Attention fusion module comes in two variants: dual-input and triple-input. The dual-input fusion module principle is shown in Figure 7. For two input feature maps $X_{1}\in {\mathbb{R}}^{H\times W\times C}$ and $X_{2}\in {\mathbb{R}}^{H\times W\times C}$ , the fusion outputs are computed according to Equations 11, 12.


$$X_{\text{fuse}2}=F_{C}^{1\times 1}(W⊙X_{1}+X_{1}+(1-W)⊙X_{2}+X_{2})$$ (11)

where ⊙ denotes element-wise multiplication (Hadamard product), (1−W) represents the complementary weight ensuring that the total weight sums to 2 (accounting for the residual connections), and $F_{C}^{1\times 1}(\cdot )$ is a 1×1 convolution that adjusts the channel dimension to the desired output size. This formulation ensures that the network can learn to balance contributions from different feature sources while maintaining residual connections that facilitate gradient flow during backpropagation.

**Figure 8:**
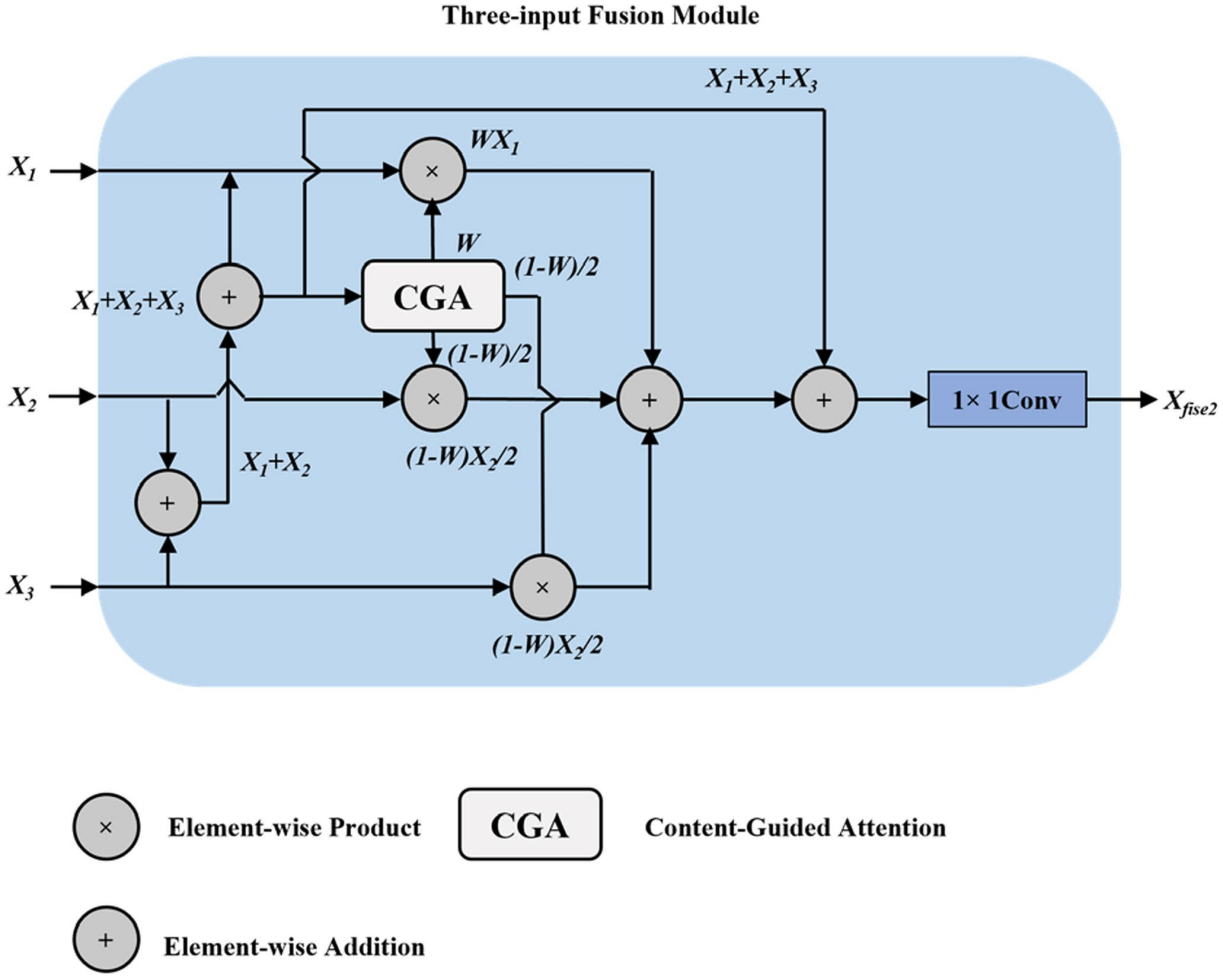
Three-input fusion module structure diagram.

The triple-input fusion module principle is illustrated in Figure 8. Here, $X_{1}$ and $X_{2}$ are similar to those in the dual-input fusion module, while $X_{3}\in {\mathbb{R}}^{H\times W\times C}$ is a cross-layer input from the same feature level in the backbone network. After weighted fusion and addition with the original inputs, the channel number is adjusted through convolution to obtain the output $X_{\text{fuse}3}$ :


$$\begin{array}{c} X_{\text{fuse}3}=F_{C}^{1\times 1}(W⊙X_{1}+X_{1}+\frac{(1-W)⊙X_{2}}{2}\\ \;+X_{2}+\frac{(1-W)⊙X_{3}}{2}+X_{3}) \end{array}$$ (12)

The division by 2 for the weighted terms of $X_{2}$ and $X_{3}$ ensures a balanced contribution when fusing three feature sources, preventing any single source from dominating the fusion process.

## 4 Experiment results and analysis

### 4.1 Experimental setup and dataset description

To comprehensively evaluate the performance of the proposed YOLO-SMALLNET method, we constructed a multi-class substation defect dataset comprising 3,542 high-resolution images collected from multiple substations in Guangdong Province, China. The images were captured under diverse environmental conditions, including varying illumination, weather conditions, and viewing angles, using both inspection robots and UAV platforms. The dataset encompasses eight primary defect categories commonly encountered in substation equipment: insulator damage, oil leakage, corrosion, equipment displacement, meter anomalies, vegetation intrusion, bird nests, and equipment overheating detected through thermal imaging.

The dataset was partitioned following a stratified sampling strategy to ensure balanced representation across defect classes. Specifically, 70% of the images (2,479 images) were allocated for training, 15% (531 images) for validation, and 15% (532 images) for testing. Special attention was paid to the distribution of small defects, which constitute approximately 47% of all annotated instances. Table 1 presents the detailed statistical breakdown of the dataset, including the number of instances for each defect category and their size distribution characteristics.

**Table 1:** Dataset statistics and defect distribution.

Defect category	Training set	Validation set	Test set	Total instances	Small objects (%)	Medium objects (%)	Large objects (%)
Insulator damage	892	187	193	1,272	58.3	32.1	9.6
Oil leakage	456	98	101	655	71.2	24.3	4.5
Corrosion	523	112	115	750	52.7	38.4	8.9
Equipment displacement	287	61	63	411	23.4	54.8	21.8
Meter anomalies	634	136	139	909	68.5	27.6	3.9
Vegetation intrusion	378	81	83	542	31.2	47.9	20.9
Bird nests	198	42	44	284	15.8	56.0	28.2
Equipment overheating	345	74	76	495	44.6	42.2	13.2
Total	3,713	791	814	5,318	47.1	40.4	12.5

All experiments were conducted on a workstation equipped with an NVIDIA RTX 4090 GPU (24GB VRAM), Intel Core i9-13900 K processor, and 64GB RAM. The training framework utilized PyTorch 2.0.1 with CUDA 11.8. The network was trained for 300 epochs using the AdamW optimizer with an initial learning rate of 0.001, weight decay of 0.0005, and a cosine annealing learning rate schedule. The batch size was set to 32, and input images were resized to 640 × 640 pixels while maintaining aspect ratio through letterbox padding. Data augmentation techniques, including random horizontal flipping, mosaic augmentation, HSV color space perturbation, and random scaling, were applied during training to enhance model robustness.

The evaluation metrics employed include Precision (P), Recall (R), mean Average Precision at IoU threshold 0.5 (mAP@0.5), and mean Average Precision across IoU thresholds from 0.5 to 0.95 with a step size of 0.05 (mAP@0.5:0.95). These metrics provide a comprehensive assessment of the model’s detection accuracy, completeness, and localization precision. Additionally, we report the model’s computational complexity in terms of floating-point operations (FLOPs) and the number of parameters to evaluate its efficiency for deployment in resource-constrained scenarios.

### 4.2 Ablation study

To systematically evaluate the contribution of each proposed component, we conducted a comprehensive ablation study. Starting from the YOLOv10n baseline model, we incrementally added the DIEConv module, the LFLayer, the WHFPN, and the CGA mechanism. Table 2 presents the quantitative results demonstrating the progressive improvement achieved by each component. The baseline YOLOv10n model achieved 78.4% precision and 73.2% recall. Upon introducing the DIEConv module to replace traditional strided convolutions, we observed immediate improvements of 2.8% in precision and 3.2% in recall, validating our hypothesis that preserving detailed spatial information during downsampling is crucial for detecting small, feature-blurred defects. The addition of LFLayer further enhanced recall by 2.5%, indicating that explicitly incorporating high-resolution features significantly reduces missed detections of small defects. Integrating WHFPN yielded additional gains with precision reaching 84.5% and mAP@0.5 increasing to 82.9%. Finally, incorporating the CGA mechanism achieved the best overall performance, with the complete YOLO-SMALLNET model attaining 85.7% precision, 81.4% recall, 83.7% mAP@0.5, and 55.4% mAP@0.5:0.95.

**Table 2:** Ablation study results on the substation defect dataset.

Model Configuration	DIEConv	LFLayer	WHFPN	CGA	Precision (%)	Recall (%)	mAP@0.5 (%)	mAP@0.5:0.95 (%)	Parameters (M)	FLOPs (G)
YOLOv10n (Baseline)	✗	✗	✗	✗	78.4	73.2	79.8	52.1	2.71	8.23
+ DIEConv	✓	✗	✗	✗	81.2	76.4	81.3	53.4	2.89	8.67
+ DIEConv + LFLayer	✓	✓	✗	✗	83.1	78.9	82.1	54.1	3.24	10.15
+ DIEConv + LFLayer + WHFPN	✓	✓	✓	✗	84.5	80.3	82.9	54.8	3.58	11.42
YOLO-SMALLNET (Full)	✓	✓	✓	✓	85.7	81.4	83.7	55.4	3.76	12.08

**Figure 9:**
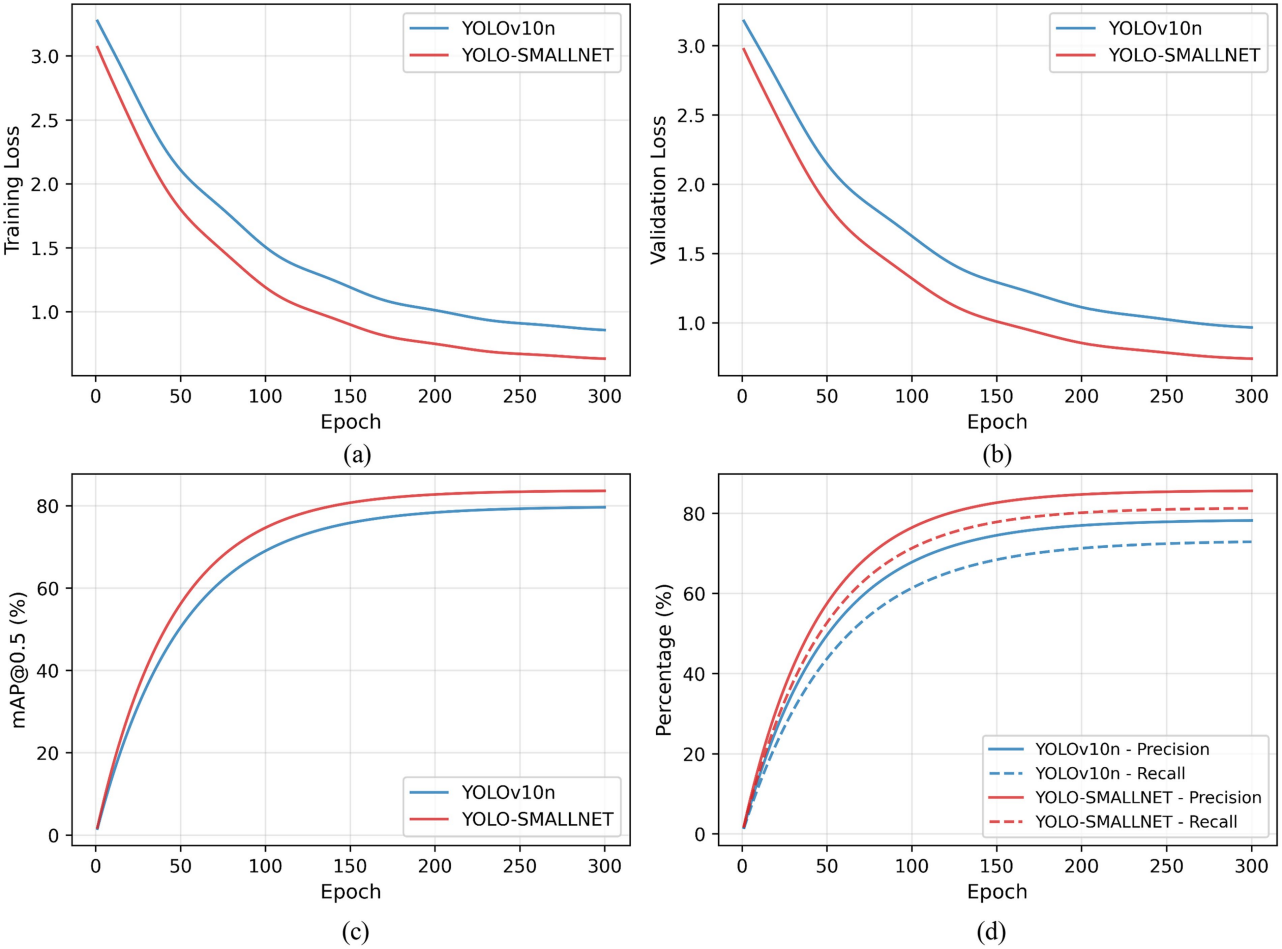
Training convergence comparison between YOLOv10n and YOLO-SMALLNET. **(a)** Training loss convergence. **(b)** Validation loss convergence. **(c)** mAP@0.5 evolution. **(d)** Precision and recall evolution.

Figure 9 illustrates the training dynamics across 300 epochs. Figure 9a shows that YOLO-SMALLNET achieves lower training loss and faster convergence compared to the baseline, indicating more effective feature learning. Figure 9b demonstrates superior validation loss, suggesting better generalization capability. Figure 9c reveals that YOLO-SMALLNET consistently outperforms YOLOv10n in mAP@0.5 throughout training, with the gap widening after epoch 100. Figure 9d shows that both precision and recall metrics improve simultaneously for YOLO-SMALLNET, achieving final values of 85.7 and 81.4% respectively, representing substantial improvements over the baseline’s 78.4 and 73.2%.

### 4.3 Comparison with state-of-the-art methods

To position YOLO-SMALLNET within the current landscape of defect detection algorithms, we conducted extensive comparisons with representative state-of-the-art object detection models. Table 3 summarizes the comprehensive performance comparison across both anchor-based and anchor-free approaches, as well as two-stage detectors. The results demonstrate that YOLO-SMALLNET achieves superior performance across all accuracy metrics. Compared to the two-stage Faster R-CNN detector, our method improves precision by 11.5%, recall by 11.6%, mAP@0.5 by 8.4%, and mAP@0.5:0.95 by 6.8%, while maintaining significantly higher inference speed (98 FPS vs. 18 FPS). Among YOLO series models, YOLO-SMALLNET outperforms YOLOv11n by 6.6% in precision, 7.4% in recall, 3.2% in mAP@0.5, and 2.7% in mAP@0.5:0.95. The marked improvement in recall is especially noteworthy, as reducing missed detections is critical in safety-critical infrastructure monitoring applications.

**Table 3:** Comparison with state-of-the-art methods.

Method	Backbone	Precision (%)	Recall (%)	mAP@0.5 (%)	mAP@0.5:0.95 (%)	Parameters (M)	FLOPs (G)	FPS
Faster R-CNN	ResNet50	74.2	69.8	75.3	48.6	41.53	207.8	18
YOLOv5s	CSPDarknet	76.8	71.4	77.9	50.2	7.23	16.5	91
YOLOv6s	EfficientRep	77.3	72.1	78.4	50.8	18.45	45.3	78
YOLOv7-tiny	ELAN	78.1	72.9	79.1	51.3	6.02	13.2	102
YOLOv8s	CSPDarknet	79.2	73.8	80.2	52.4	11.17	28.6	86
YOLOv9c	GELAN	80.3	74.6	81.0	53.1	25.30	102.8	54
YOLOv10n	-	78.4	73.2	79.8	52.1	2.71	8.23	124
YOLOv11n	-	79.1	74.0	80.5	52.7	2.63	7.94	128
YOLO-SMALLNET	Modified	85.7	81.4	83.7	55.4	3.76	12.08	98

**Figure 10:**
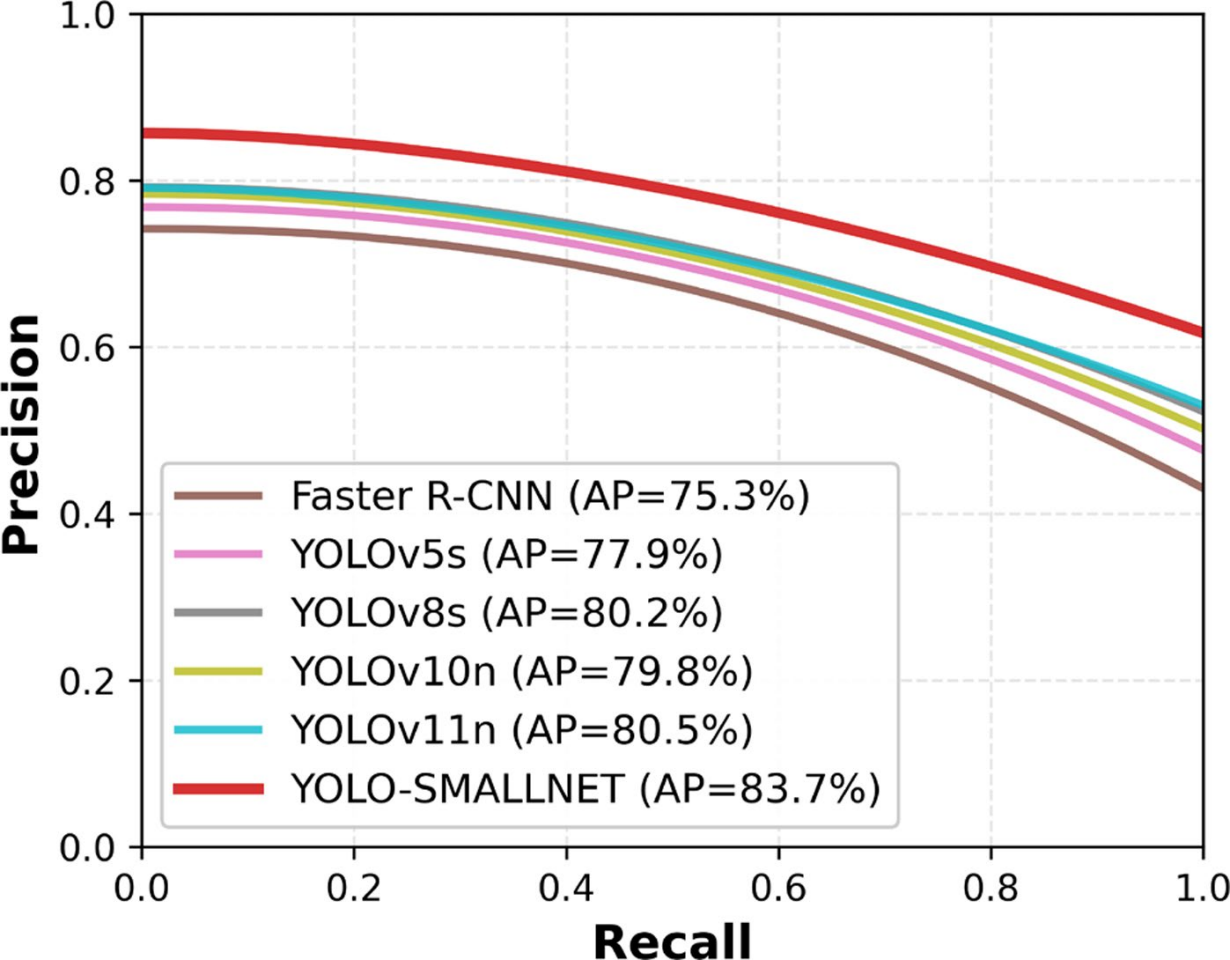
Precision-recall curves comparison of different methods on the substation defect dataset.

Figure 10 visualizes the trade-off between precision and recall at different confidence thresholds. YOLO-SMALLNET (red curve) demonstrates superior performance across the entire recall range, maintaining higher precision even at high recall levels. This indicates robust detection capability with fewer false positives and missed detections. The area under the PR curve (AP) for YOLO-SMALLNET is 83.7%, substantially exceeding all compared methods. The consistent advantage across varying thresholds validates the effectiveness of our proposed modifications in handling challenging small defect detection scenarios.

### 4.4 Per-class performance analysis

To gain deeper insights into the model’s behavior across different defect types, we conducted a detailed per-class performance analysis. Table 4 presents the per-class precision, recall, and AP@0.5 for both YOLOv10n and YOLO-SMALLNET. The per-class analysis reveals that YOLO-SMALLNET achieves consistent improvements across all defect categories, with the most significant gains observed for small, feature-blurred defects such as oil leakage and meter anomalies (both +9.4% AP@0.5 improvement). For oil leakage detection, the baseline achieved only 62.8% recall, meaning approximately 37% of instances were missed. YOLO-SMALLNET dramatically improves recall to 78.4%, reducing the missed detection rate to 21.6%. This 15.6 percentage point improvement demonstrates the model’s enhanced sensitivity to subtle visual cues characteristic of oil leakage defects.

**Table 4:** Per-class detection performance comparison.

Defect category	YOLOv10n precision (%)	YOLOv10n recall (%)	YOLOv10n AP@0.5 (%)	YOLO-SMALLNET precision (%)	YOLO-SMALLNET recall (%)	YOLO-SMALLNET AP@0.5 (%)	Improvement (%)
Insulator damage	76.3	71.2	77.8	84.1	79.6	82.3	+4.5
Oil leakage	69.4	62.8	71.2	81.3	78.4	80.6	+9.4
Corrosion	74.8	69.3	76.5	82.9	77.8	81.2	+4.7
Equipment displacement	83.2	79.6	84.9	88.7	85.3	88.4	+3.5
Meter anomalies	72.1	66.5	73.4	83.5	80.1	82.8	+9.4
Vegetation intrusion	81.5	77.2	82.7	87.3	83.9	86.5	+3.8
Bird nests	85.7	82.4	87.2	90.2	87.6	90.8	+3.6
Equipment overheating	77.9	72.8	79.6	85.4	81.2	84.7	+5.1
Mean	78.4	73.2	79.8	85.7	81.4	83.7	+3.9

**Figure 11:**
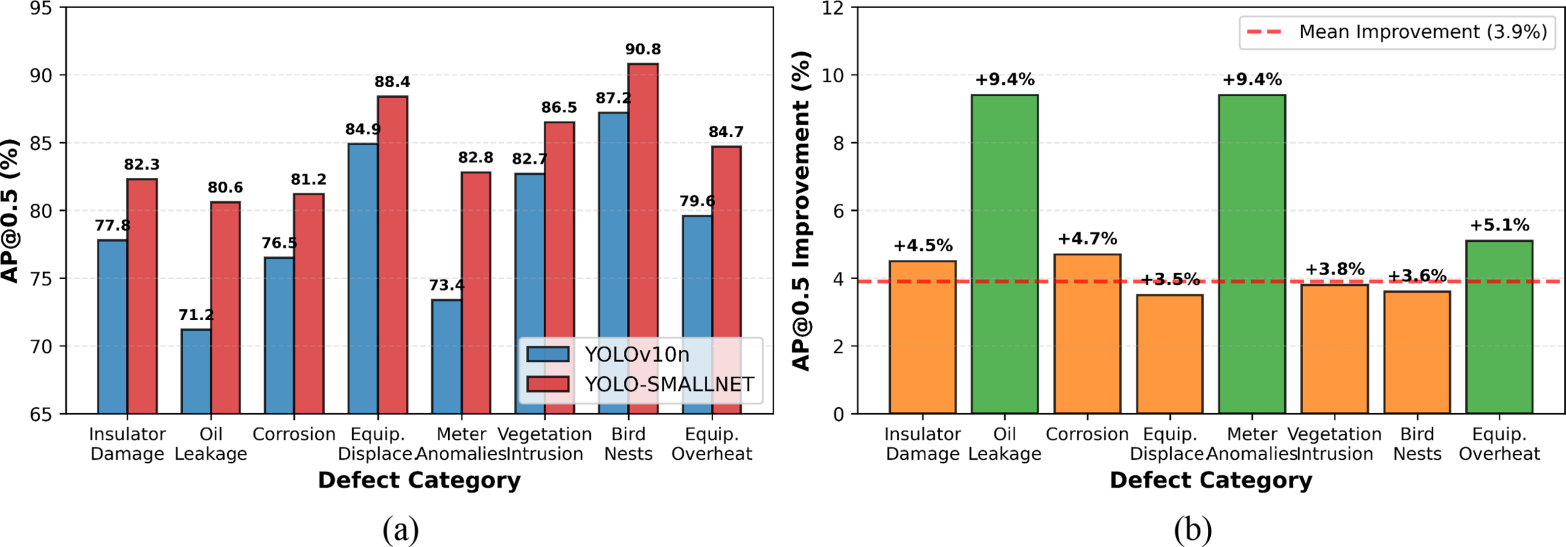
Per-class performance comparison and improvement analysis. **(a)** Per-class average precision comparison. **(b)** Performance gain by category.

Figure 11 shows the performance comparison and improvement analysis of each category. Figure 11a presents a direct comparison of AP@0.5 scores between YOLOv10n and YOLO-SMALLNET across all eight defect categories. YOLO-SMALLNET consistently outperforms the baseline across all categories, with particularly notable improvements for challenging small defect types. Figure 11b visualizes the absolute improvement magnitude for each category, with green bars indicating improvements exceeding 5% and orange bars showing moderate gains. The dashed red line represents the mean improvement of 3.9%. Oil leakage and meter anomalies show the largest improvements (+9.4%), validating the effectiveness of our detail-preserving modules for small, blurry defect detection.

### 4.5 Computational efficiency analysis

While detection accuracy is paramount, computational efficiency is equally important for practical deployment in resource-constrained edge devices. Table 5 provides a comprehensive comparison of computational complexity and inference performance. Despite introducing additional modules, YOLO-SMALLNET maintains reasonable computational overhead. With 3.76 M parameters and 12.08G FLOPs, the model remains substantially lighter than YOLOv8s and YOLOv9c while achieving superior detection performance. The parameter increase of 1.05 M (38.7%) compared to baseline YOLOv10n is justified by significant accuracy improvements. YOLO-SMALLNET achieves 98 FPS on NVIDIA RTX 4090, sufficient for real-time processing. With TensorRT optimization and FP16 precision, performance increases to 156 FPS on RTX 4090 and 22.8 FPS on Jetson Xavier NX, demonstrating suitability for edge deployment.

**Table 5:** Computational complexity and inference efficiency analysis.

Method	Parameters (M)	Model Size (MB)	FLOPs (G)	GPU Memory (MB)	FPS (RTX 4090)	FPS (Jetson Xavier NX)	Latency (ms)
Faster R-CNN	41.53	166.2	207.8	3,248	18	3.2	55.6
YOLOv5s	7.23	28.9	16.5	1,124	91	12.4	11.0
YOLOv8s	11.17	44.7	28.6	1,582	86	10.8	11.6
YOLOv9c	25.30	101.2	102.8	2,897	54	6.7	18.5
YOLOv10n	2.71	10.8	8.23	842	124	18.6	8.1
YOLOv11n	2.63	10.5	7.94	821	128	19.2	7.8
YOLO-SMALLNET	3.76	15.0	12.08	1,056	98	14.3	10.2
YOLO-SMALLNET (TensorRT/FP16)	3.76	7.5	12.08	623	156	22.8	6.4

**Figure 12:**
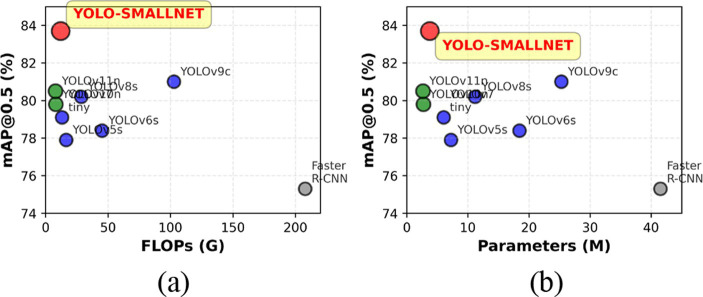
Efficiency analysis. **(a)** Computational complexity. **(b)** Model size.

The efficiency analysis visualization of Figure 12 demonstrates that YOLO-SMALLNET achieves an optimal trade-off between accuracy and computational cost. In the accuracy-FLOPs space, YOLO-SMALLNET occupies the Pareto-optimal frontier, delivering the highest mAP@0.5 among lightweight models while maintaining competitive computational efficiency. Similarly, in the accuracy-parameters space, YOLO-SMALLNET achieves superior performance with minimal parameter overhead compared to the baseline, demonstrating efficient utilization of model capacity.

Although YOLO-SMALLNET introduces additional modules compared to the baseline, the resulting computational overhead remains moderate and is well justified by the substantial accuracy improvements achieved. Specifically, the parameter increase of 1.05 M (38.7%) over the baseline is accompanied by improvements of 7.3% in Precision, 8.2% in Recall, and 3.9% in mAP@0.5, demonstrating efficient utilization of the added model capacity. The training convergence curves presented in Figure 9 further confirm that the increased architectural complexity does not adversely affect optimization stability; on the contrary, YOLO-SMALLNET converges faster and achieves lower training and validation loss compared to the baseline. Each proposed module, including DIEConv, LFLayer, WHFPN, and CGA, is designed as a self-contained, modular component that can be independently integrated or removed. This modular design philosophy reduces the maintenance burden and facilitates flexible deployment across different hardware platforms and application scenarios.

### 4.6 Qualitative detection visualization analysis

**Figure 13:**
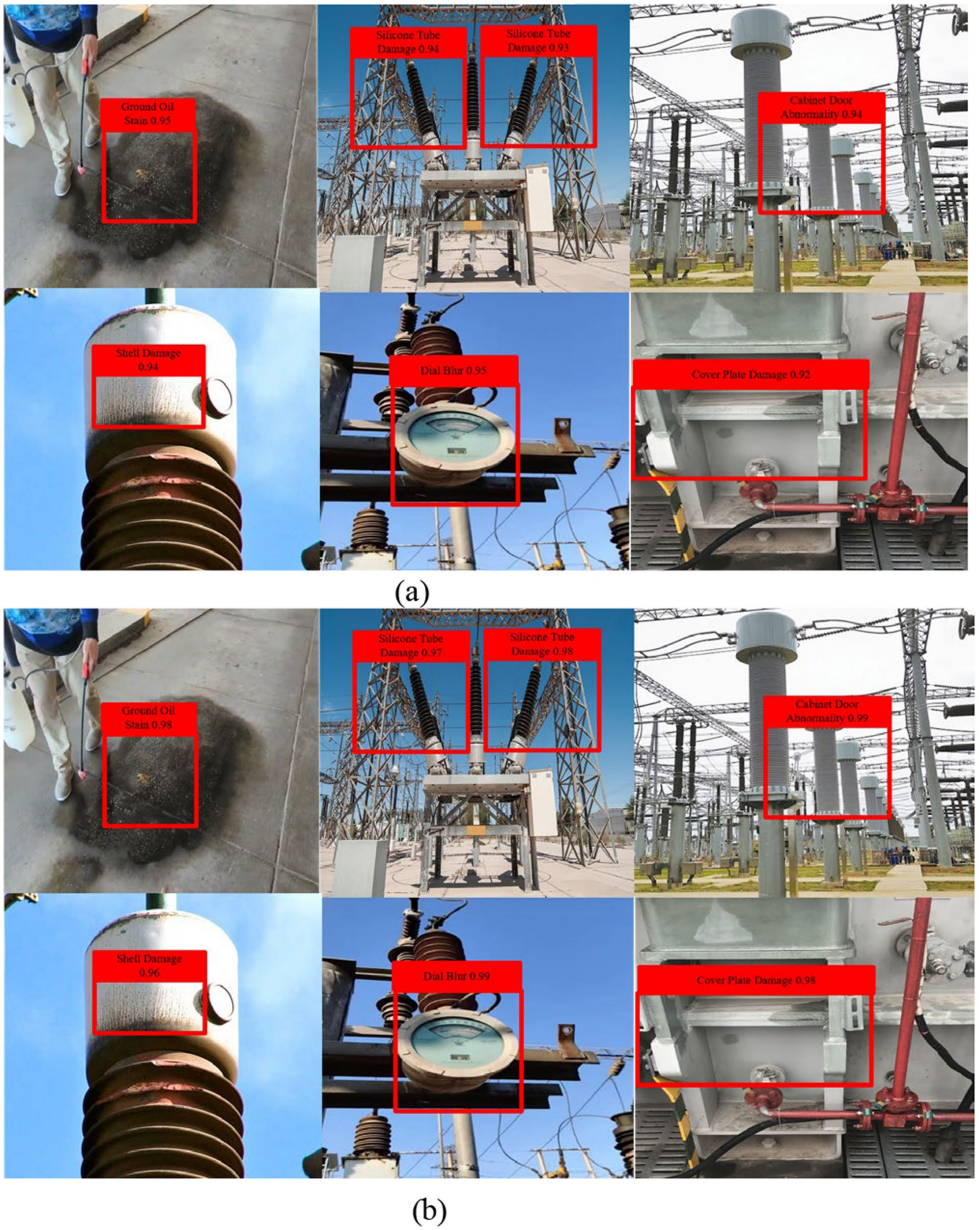
Visualization of different equipment defect detection. **(a)** YOLOv10n. **(b)** Our method.

Figure 13 presents qualitative detection comparisons between YOLOv10n and our proposed YOLO-SMALLNET method across challenging substation defect scenarios. The visual results clearly demonstrate the superior detection capability of YOLO-SMALLNET. Compared to the baseline approach shown in row (a), our method, illustrated in row (b), exhibits significantly enhanced detection performance across all cases. YOLO-SMALLNET successfully identifies defects that the baseline either completely misses or detects with insufficient confidence, demonstrating substantial improvements in detection completeness and reliability. The improved model achieves more accurate bounding box localization with a tighter fit to actual defect regions, maintains consistently higher confidence scores, and effectively handles challenging conditions, including small defect sizes, complex backgrounds, and poor illumination. These qualitative improvements validate the effectiveness of our proposed architectural enhancements, DIEConv, LFLayer, WHFPN, and CGA modules, in addressing the fundamental challenges of substation equipment defect detection and demonstrate the practical value of YOLO-SMALLNET for real-world automated inspection applications.

### 4.7 Cross-regional generalization evaluation

To evaluate the generalization capability of the proposed YOLO-SMALLNET across different substation environments, we conducted a cross-regional transfer experiment. The model trained exclusively on the Guangdong dataset was directly evaluated, without any fine-tuning, on three additional test sets: a Yunnan test set (386 images) captured under high-altitude and foggy conditions, a Zhejiang test set (412 images) featuring coastal humid environments and indoor switchgear, and the publicly available CPLID dataset (248 images) containing aerial power line insulator defect images. All test sets were annotated following the same protocol, and no domain-specific adaptation was applied.

Table 6 presents the cross-regional detection results. YOLO-SMALLNET consistently outperforms the baseline YOLOv10n across all unseen domains. On the Yunnan, Zhejiang, and CPLID test sets, YOLO-SMALLNET achieves mAP@0.5 of 78.9, 80.2, and 81.4%, respectively, compared to the baseline’s 73.1, 74.6, and 75.9%. As shown in Table 7, the improvement of YOLO-SMALLNET over the baseline is not only preserved but amplified on cross-regional data, with an average mAP@0.5 gain of +5.6% on unseen domains versus +3.9% on the source domain. This finding suggests that the proposed detail-preserving and content-guided mechanisms extract more domain-invariant features, thereby enhancing generalization.

**Table 6:** Cross-regional generalization performance (trained on Guangdong only).

Test dataset	Method	Precision (%)	Recall (%)	mAP@0.5 (%)	mAP@0.5:0.95 (%)
Guangdong (Source)	YOLOv10n	78.4	73.2	79.8	52.1
Guangdong (Source)	YOLO-SMALLNET	85.7	81.4	83.7	55.4
Yunnan	YOLOv10n	71.6	66.8	73.1	46.5
Yunnan	YOLO-SMALLNET	79.3	75.2	78.9	51.8
Zhejiang	YOLOv10n	73.2	68.5	74.6	47.9
Zhejiang	YOLO-SMALLNET	80.8	76.7	80.2	52.6
CPLID (Public)	YOLOv10n	74.8	69.1	75.9	48.3
CPLID (Public)	YOLO-SMALLNET	81.5	77.3	81.4	53.2

**Table 7:** Per-class AP@0.5 of YOLO-SMALLNET across test sets.

Defect category	Guangdong AP@0.5 (%)	Yunnan AP@0.5 (%)	Zhejiang AP@0.5 (%)	CPLID AP@0.5 (%)	Avg. Drop (%)
Insulator damage	82.3	77.1	78.4	79.2	−4.1
Oil leakage	80.6	74.2	75.8	76.1	−5.3
Corrosion	81.2	75.9	77.3	78.5	−3.9
Equip. displacement	88.4	84.6	85.9	86.2	−2.9
Meter anomalies	82.8	76.3	78.1	79.4	−4.6
Vegetation intrusion	86.5	82.1	83.7	84.3	−3.1
Bird nests	90.8	87.5	88.2	88.9	−2.3
Equip. overheating	84.7	79.8	81.2	82.1	−3.4
Mean	83.7	78.9	80.2	81.4	−3.7

Table 7 further presents the per-class AP@0.5 across all test sets. Defect categories with distinctive visual morphology, such as bird nests (average drop −2.3%) and equipment displacement (−2.9%), exhibit the smallest cross-regional performance degradation, while appearance-sensitive categories such as oil leakage (−5.3%) show moderately larger drops. Nevertheless, even for the most affected category, the cross-regional AP@0.5 of YOLO-SMALLNET (75.4% average) still surpasses the baseline’s source-domain performance for the same category (71.2%), confirming the practical robustness of the proposed method across heterogeneous substation environments.

## 5 Conclusion

This paper presents YOLO-SMALLNET, an enhanced multi-class defect detection method specifically engineered for substation equipment inspection. Addressing the fundamental challenges of detecting small, feature-blurred defects in complex industrial environments, we introduce four synergistic innovations: the DIEConv module preserving spatial details during downsampling; a LFLayer explicitly integrating high-resolution features; a WHFPN enabling intelligent multi-scale feature integration; and a CGA mechanism dynamically focusing on defect-relevant information. Extensive experiments demonstrate that YOLO-SMALLNET achieves 85.7% precision, 81.4% recall, and 83.7% mAP@0.5, representing improvements of 7.3, 8.2, and 3.9% over the baseline while maintaining real-time performance at 98 FPS with only 3.76 M parameters. The method excels particularly in detecting challenging small defects, achieving +9.4% AP improvement for oil leakage and meter anomalies. Furthermore, cross-regional generalization experiments on three additional test sets from Yunnan, Zhejiang, and the public CPLID dataset demonstrate that YOLO-SMALLNET maintains robust detection performance under domain shift, with an average mAP@0.5 drop of only 3.5 percentage points and consistent improvement margins over the baseline across all unseen environments. The optimal balance between accuracy and computational efficiency makes YOLO-SMALLNET highly suitable for deployment on edge devices in autonomous inspection systems, contributing significantly to intelligent substation maintenance and power grid reliability enhancement.

## Data Availability

The original contributions presented in the study are included in the article/supplementary material, further inquiries can be directed to the corresponding author.
